# Latent tuberculosis infection and biologic agents other than TNF-α inhibitors: “over-screening and over-treatment?”

**DOI:** 10.36416/1806-3756/e20240277

**Published:** 2024-09-16

**Authors:** Ana Paula Santos, Fernanda Carvalho de Queiroz Mello

**Affiliations:** 1. Instituto de Doenças do Tórax - IDT - Universidade Federal do Rio de Janeiro - UFRJ - Rio de Janeiro (RJ) Brasil.; 2. Hospital Universitário Pedro Ernesto, Universidade do Estado do Rio de Janeiro - UERJ - Rio de Janeiro (RJ) Brasil.

The advent of biological therapy to treat many conditions, including autoimmune diseases, asthma, and even cancer, has changed the prognosis of millions of patients, decreasing morbidity and mortality and increasing quality of life.^1^ However, because of their mechanism of action, biological therapy can increase the risk of some infections, including tuberculosis.^2^

Latent tuberculosis infection (LTBI), or tuberculosis infection (TBI), as defined in the original article published by Sultana et al.^3^ in this issue of the Journal, has a risk of reactivation increased by almost 30 times in patients using TNF-α inhibitors.^2^ This happens because such inhibitors block one of the most important cytokines responsible for the integrity of the tuberculous granuloma.^4^ However, it is still unclear whether biological therapy with other agents are associated or not with the risk of turning LTBI into active tuberculosis.^5^ Additionally, clinical practice is largely heterogeneous, given different scenarios and tuberculosis burdens, which hinders the generalization of guidelines.

Sultana et al.^3^ bring a very interesting and timely topic by describing the different clinical practices regarding the approach to TBI worldwide and by evaluating if they are in line with their respective national or international guidelines. In that study,^3^ 163 responders in 27 countries completed the survey. According to the authors, “TBI screening rates in patients treated with TNF-α inhibitors were high, especially for older TNF-α inhibitors. Most participants supported TBI screening in patients treated with B- or T-cell inhibitors but not in those treated with interleukin inhibitors. Guideline awareness was higher for TNF-α inhibitors than for other biologic classes.” They came to the conclusion that there was a “tendency to recommend TBI screening in patients treated with biologics not known to be associated with an increased risk of TBI” and, as a result, “there is a potential risk of over-screening and over-treatment of TBI, potentially causing harm, in patients treated with biologics other than TNF-α inhibitors.” Finally, they conclude that there is a need to investigate the risk of TBI associated with biologics and to develop guidelines to address the spectrum of TBI risk across all types of biologics.

After the initial increasing number of active tuberculosis in the beginning of 2000s associated with TNF-α inhibitors,^6^ the scientific community, including physicians, researchers, and even the pharmaceutical industry, became really concerned about this serious adverse event related to these drugs. Since then, each biological therapy approved for use has been considered to be likely to increase active tuberculosis risks, with regard to their different mechanisms of action and potential interference in tuberculosis immunopathogenesis. This concern, although proven to be minimal, pushed all of us to recommend screening and TBI treatment for every patient under biological treatment.^5^ ^,^ ^7^ Now, some years after clinical use, more expertise with all these biological drugs and new researches have shown the safety of biologics other than TNF-α inhibitors. It is time to ask if we still should continue screening and treating those with low or even no risk of tuberculosis reactivation.^5^

Indeed, there is no doubt regarding the over-screening and treatment, especially considering the possible adverse effects related to the TBI treatment, known not to be free from severe complications such as hepatotoxicity. However, TBI is a recurring topic on the “End TB Strategy”^8^ agenda, aiming to contribute to achieving the difficult targets of reducing the incidence and mortality of tuberculosis. A backward step, reducing preventive measures, should be discussed. The argument for this concern about TBI treatment in patients on biological therapy relies on the fact that the indication is not only related to the mechanism of action of these drugs, but also to the immunosuppressive condition inherited by autoimmunity. Since the beginning of the last century, studies have shown a higher incidence of active tuberculosis in patients with rheumatoid arthritis, psoriasis, and inflammatory bowel disease, for example.^9^ Besides that, there is the possibility of combining these non-TNF-α inhibitor biological therapy with other non-biological disease modifying antirheumatic drugs, which also increase the risk of TBI activation, such as corticosteroids, methotrexate, and leflunomide.^10^ Furthermore, patients can have more than one condition that indicate TBI screening.^11^

Differences in how and when to screen for TBI according to the results of Sultana’s research are highlighted. Although interferon gamma release assays and tuberculin skin tests were correctly mentioned in different percentages, almost 40% of the respondents were not in favor of performing chest X-rays in all patients during screening, regardless of the presence of symptoms or test results.^3^ This should be a reflection of the different recommendations on screening for LTBI around the world. In addition, the responses on when to repeat screening were heterogeneous, probably also reflecting the different origins of the respondents and their different practices according to the tuberculosis burden at their place of practice.^11^ ^,^ ^12^

Although there are well-established indications for TBI screening and treatment, another concern is related to clinical practitioner adherence to guidelines and recommendations proposed by different official societies and organizations.^3^ In the study by Sultana et al.,^3^ current practice did not always align with national guidelines regarding screening for TBI in patients under immunosuppressive treatment. Also, in some countries, the national guidelines were not updated, which could explain such divergences. Standardized conducts are important, especially in continental and medium-to-high burden countries such as Brazil.

There are several issues to be taken into consideration when deciding to screen and treat TBI in patients on immunosuppressive therapy. When it comes to *Mycobacterium tuberculosis*, one size does not fit all, and many aspects must be relevant. First, the diagnostic methods available for TBI are not perfect. In addition to their expected false negative results, which any test can have, until today, we have no test to diagnose reinfection after treatment, which can be common in high burden tuberculosis countries.^12^ Second, we should take into consideration TBI treatment regimens and risks of drug interactions. Finally, we must know the tuberculosis prevalence in different scenarios, the patients’ comorbidities, and the risks of adverse events.^11^

In times of so many questions, Sultana et al ^3^ hit the target bringing this discussion and making us understand the urgent need for new researches to assess the risk of tuberculosis activation according to the immunosuppressive treatment, and mostly, for updated guidelines to address the spectrum of TBI in specific populations and different scenarios.
